# The Appearance of The Infraorbital Canal and Infraorbital Ethmoid (Haller's) Cells on Panoramic Radiography of Edentulous Patients

**DOI:** 10.1155/2018/1293124

**Published:** 2018-07-08

**Authors:** Esra Yesilova, Ibrahim Sevki Bayrakdar

**Affiliations:** Oral and Maxillofacial Radiology Department, Faculty of Dentistry, Eskişehir Osmangazi University, Eskişehir, Turkey

## Abstract

*Objectives. *The aim of the study is to detect the prevalence and the characteristics of infraorbital canal and Haller's cells on panoramic radiography of edentulous patients.* Methods. *The study group comprised 291 panoramic radiographs of edentulous patients. Radiographs were interpreted for the visibility and characteristics of infraorbital canal and Haller's cells. For classification of infraorbital canal, a method based on the image characteristics of the border of the canal (Types I, II, and III) was used. Haller's cells were grouped according to the number and the shape of loculations.* Results. *Infraorbital canal was observed in 246 (84.6%) radiographs. The most prevalent of the observed canals were Type III for both sides (39.9 % for right and 32.3% for left side). The visibility of Haller's cells was 23.7%. The frequencies of Haller's cells' visibility were approximately equal for both genders. There is no significant difference between genders for the visibility of infraorbital canal and Haller's cells.* Conclusions. *The surgeons, implantologists, and radiologists should take into consideration infraorbital canal and Haller's cell for planning implant surgery of maxillary anterior region and undefined orofacial pain for edentulous patients.

## 1. Introduction

The infraorbital region is a passage between cranial fossa, osteomeatal complex, orbits, and the maxillary dental segment. The infraorbital nerve is one of the major anatomical structures of this region. It is a division of maxillary nerve, extending from the inferior orbital fissure to infraorbital foramen throughout maxillary sinus in the infraorbital canal/groove complex (ICG/C) [1–3].

There have been numerous radiological [1–5] and anatomical [6–10] studies about the infraorbital nerve's dimensions and types in both living persons and cadavers/skulls. Some authors reported anatomical variations [11, 12] and classifications [2–5] of ICG/C. With the introduction of three-dimensional (3D) techniques to clinical practice, neighbouring anatomical variations were noticed, which affected the classifications [11]. Haller's cells are neighbouring structures of ICG/C. They are called either orbitoethmoidal cells or maxilloethmoidal cells. The name* infraorbital ethmoid cell* is more proposed to describe the site and the emergence of these objects. Haller's cells may be different in size, number, and shape [13]. When enlarged, they can significantly constrict posterior aspect of the infundibulum. These entities can be associated with symptoms of rhinosinusitis such as orofacial pain, headache, and impaired nasal breathing [14]. Isolated Haller's cell mucocele cases were reported [15, 16]. Therefore the presence of Haller's cells is clinically significant. Even with rhinoscopy, it is not easy to observe Haller's cells because of their location, which may be near or extend into the infraorbital canal. Radiology is indispensable for diagnosis [15].

Panoramic radiography is a practical technique that presents an image of a large area including midface bones (nasal fossa, orbital fossae, and maxillary sinus) and teeth. Patients easily tolerate the application of this technique. Despite some structural superimpositions and magnifications [17], plain radiography is still the first choice for evaluation with a low radiation dose. It is commonly used to examine dentate and edentate jaws. In a review of related literature, Scarfe's [5] classification seems to be the only one on evaluating the infraorbital canal with panoramic radiography. It is a simple and useful classification that will be described in more detail in the* Methods and Materials* of this paper. Haller's cell has also been demonstrated in panoramic images by some researchers [13, 14, 18].

The rehabilitation of edentulous patients with implant-supported prosthesis now has an important role as a treatment modality in dental practice. While patients have expressed increased demand for implants, the amount and density of bone, metabolic bone disorders, and variations of adjacent anatomic structures are limiting factors. Sinus-lifting techniques are needed to deal with atrophied alveolar ridges [19]. Surgical techniques require knowing region and its possible variations well. In upper jaw and midface surgeries, the knowledge of the infraorbital canal anatomical structure minimizes the damage to nerves in surgical approach of orbits, zygomatic process, and maxillary bone [3].

Other than prosthetic rehabilitation, edentulous patients also consult dentists for diagnosis and treatment of orofacial pain [20]. Sometimes patients have to visit different departments such as neurology, ear-nose-throat, and maxillofacial surgery to find the source of undefined pain. Diseases of anatomical variants may play a role in this kind of pain in edentulous patients [18].

The aim of this retrospective study is to detect the prevalence and the characteristics of infraorbital canal and Haller's cells through panoramic radiography of edentulous patients.

## 2. Materials and Methods

### 2.1. Study Design

The study group comprised 291 diagnostically acceptable panoramic radiographs of edentulous patients, randomly selected from the archive of Oral and Maxillofacial Radiology Department and evaluated retrospectively. The age range of patients was 38–88 years.

No ethical approval was required because no additional exposure of radiation was applied to the patients beyond routine diagnostic purpose in this retrospective study.

### 2.2. Data Collection

All radiographs were taken with a digital panoramic X-ray machine (Promax, Planmeca, Helsinki, Finland) (64 kV, 6 mA, 16 s). First, 60 radiographs were viewed on the same computer (MacBook Pro., China) and independently interpreted under optimal lighting conditions by two oral and maxillofacial radiologists. Interrater agreement was calculated. Kappa values were found with perfect agreement, 0.86 for Haller's cells and 0.89 for infraorbital canal, so all of the radiographs were interpreted with common agreement of two oral and maxillofacial radiologists for the visibility and characteristics of infraorbital canal and Haller's cells.

#### 2.2.1. The Criteria for Classifying Infraorbital Canal

**Figure 1:**
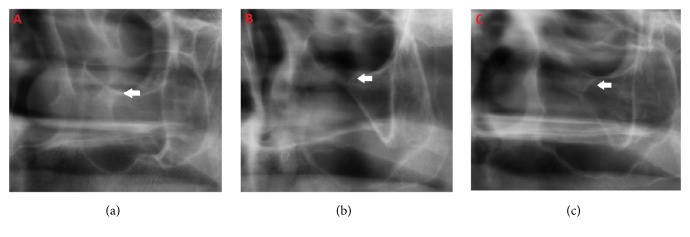
The cropped panoramic radiographic images show the types of infraorbital canals: (a)* Type I*, corticated both orbit and antrum parts corticated, (b)* Type II*, both orbit and antrum parts without cortication, and (c)* Type III*, orbital part without cortication, antrum part corticated.

The radiographic characteristics of the border of the canals were grouped according to the classification of* Scarfe* [5]* et al. *(Figure 1).

*Type I* **:** Both the borders of the orbital and the antrum parts of the canal are radiopaque

*Type II* **:** Both the borders of the orbital and the antrum parts of the canal are invisible with no linear radiopacity

*Type III* **:** The orbital part of the canal is radiolucent with no linear radiopacity; the antrum part of the canal is radiopaque with linear radiopacity

### 2.3. Infraorbital Ethmoid Cell (Haller's Cell)

**Figure 2:**
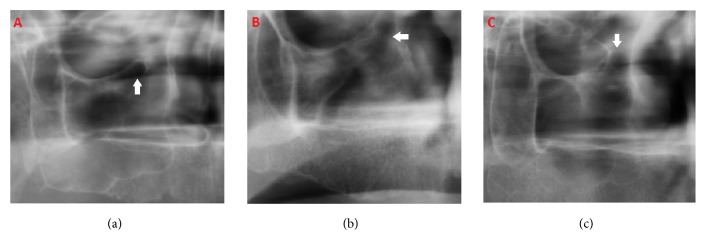
Types of infraorbital ethmoid cells (Haller's cells): (a)* unilocular,* (b)* multilocular*, and (c)* unilocular with septae*.

Haller's cells were grouped according to the number and the shape of loculations as multilocular, unilocular with septae (clustered minor locules), or unilocular (without septae) (Figure 2).

The visibility of the canal and Haller's cells grouped as* unilateral, bilateral, *and* no appearance*. Identification of Haller's cell was made according to criteria of* Ahmad et al.* [13] as follows:

(1) Well-defined, round, tear-drop shaped radiolucency, single or multiple, unilocular or multilocular, with a smooth border, which may or may not be corticated

(2) Located medial to infraorbital foramen

(3) All or most of the border in the panoramic section being visible

(4) The inferior border of the orbit lacks cortication or remains indistinguishable in areas superimposed by this entity.

### 2.4. Statistical Analysis

Data were analyzed statistically with IBM SPSS Statistics 20.0 using frequencies/percentages, descriptive statistics, cross table, and *χ*^2^ test to obtain the findings.

## 3. Results

The study group consisted of 291 radiographs from 103 males and 188 females, age ranging from 38 to 88 (mean 63.63±10.113).

### 3.1. Infraorbital Canal

Infraorbital canal was visible in 246 (84.5%) of radiographs (Table 1). In 203 cases (83%) it was observed bilaterally; in 43 cases (17%) it was observed unilaterally. It was observed in 83.5% of male patients (16% unilateral, 84% bilateral) and 85.1% of female patients (18% unilateral, 82% bilateral). Most of the observed canals were Type III for both sides (50% right and 44% left side). For the right side, 85 cases were Type I, 30 cases were Type II, and 116 cases were Type III. For the left side, 81 cases were Type I, 39 cases were Type II, and 94 cases were Type III. In 63 cases, a Type III canal was observed bilaterally.

There was no significant difference between genders for the visibility of infraorbital canal (p=0.877).

### 3.2. Haller's Cell

The prevalence of Haller's cells was 23.7%, with 69 cases showing 88 Haller cells. Haller's cells were frequently (72.5%) observed unilaterally. In 69 patients with Haller's cells, 50 were unilateral (equal for right and left sides) and 19 were bilateral. For the right side, 36 presentations were unilocular, 4 were multilocular, and 4 were unilocular with septae (clustered). For the left side, 37 presentations were unilocular, 1 was multilocular, and 6 were unilocular with septae (clustered) (Table 2). Most of the 19 bilaterally observed cells were unilocular, as in 14 cases.

Haller's cells were observed in 25.2% of cases with an observed infraorbital canal. In 16 cases, both Haller's cells and the infraorbital canal were observed bilaterally. Haller's cells were dominantly observed with Type III and I infraorbital canals. However there was no relationship between presence of Haller's cells and infraorbital canal types (p=0.162).

Haller's cells were observed in 24.3% of male patients and 23.4% female patients. There was no significant difference between genders for the visibility of Haller's cells (p=0.871).

## 4. Discussion

This study evaluated the infraorbital canal and infraorbital ethmoid cells in panoramic radiography of edentulous patients concomitantly.

Studies of the infraorbital canal are in great demand within researchers working on this region because of its clinical importance. Morphometric analyses of the infraorbital canal are made on dry skulls [6–8, 10] and cadavers [9]. For surgical planning, the course of canal through the sinus and the relationship of canal with maxillary sinus septae were studied with 3D imaging systems [3, 4]. Existing studies have all focused on possible anatomical variations to improve clinical applications [21].

Panoramic radiography creates two-dimensional (2D) images inferior to 3D systems. Nevertheless, there are some reasons for using panoramic radiography to distinguish the infraorbital canal. It enables the viewer to diagnose anatomical structures of a large area with single dose of low radiation. In addition, the projection angle of orthopantomography allows observation of the scope of infraorbital canal. As such, it is valuable to make this evaluation with this commonly used imaging technique [5].

Regardless of patient gender, canal was most commonly observed bilaterally. The first and second most observed types were Types III and I, respectively. The observation rate of the canals in panoramic radiography and the frequency of the types of the infraorbital canals are highly compatible with Scarfe's [5] results.

Haller's cells have been studied for different purposes, such as the prevalence and morphologic features of the cells and the role of the cells in rhinosinusitis. Some isolated Haller's cells pathologies [15, 16] occur in the literature. The reported incidence of Haller's cells varies according to imaging techniques, number of patients, and probably racial differences. This study was limited to a special population with an age group quite different from other studies. In other studies, a wide range was selected.

Our study found noticeable unilateral location and unilocular morphology of the cells and no relationship with gender. These findings are consistent with the findings of previous studies [14, 18]. The prevalence of Haller's cell was founded between 16 and 38.2% in panoramic radiographic studies [13, 14, 18]. Our study's prevalence was within that range, at 23.7%.

Panoramic radiography plays an important role in the determination of the infraorbital canal and Haller's cells in a considerable number of cases in dental practice. However, diagnostic value of panoramic radiography is limited because of inherited disadvantages. 3D images with thinner slices reveal cells more sensitively. But infraorbital canal and Haller's cells are anatomic variations [13]. Unless it is required to diagnose a suspicious pathology, there is no need for these sensitive images with their high radiation doses [13, 22].

When Haller's cells are inflamed, midface hypoesthesia may be experienced. In these cases, infraorbital nerve pathologies should be differentiated [15]. As mentioned above, the relationship between septae and the infraorbital canal has been studied with 3D techniques, notably in a study by Ference et al. [4] that included Haller's cells in the classification of these relationships. In our study, Haller's cells were observed in 25.2% of cases with an observed infraorbital canal, and Haller's cells were found most commonly with Type III and I infraorbital canals that had corticated borders in the maxillary sinus. It is possible to think that Ference's Type 3 canal, defined as the “descending” type that passes through the septa or is associated with Haller's cells, is compatible with our findings. The canal type associated with Haller's cells comes to an end in an infraorbital foramen that is 2.9 mm inferiorly localized compared to other types. This may be an effective guide for surgeons in clinical applications when 3D images are absent.

Classifications of the canal with computed tomography (CT) bring better visualization of the course of the canal and relationships [3, 4] than 2D images. In a recently published article [1], cone beam computed tomography (CBCT) was used for evaluation of the infraorbital canal through the maxillary sinus. Results of this CBCT study indicate that this technique may take place of CT, because of the low dose of radiation. We think that evaluation of panoramic radiography accurately will guide us for the next step toward use of 3D systems.

CBCT studies of Haller's cells have focused on the prevalence and role of these cells in rhinosinusitis [23–25]. Measurements of the infraorbital canal [26] and foramen [26, 27] show that CBCT has the potential to present this region in detail with low dose radiation. CBCT can also demonstrate relationships between different anatomical studies, as different classifications may be combined to establish a useful method for practice.

Magnetic resonance imaging (MRI) is not the first choice for drawing corticated anatomic structures [28] despite its advantage of lacking ionizing radiation. The excellent soft tissue resolution of MRI is utilized to visualize enlargement of the infraorbital nerve in orbital lymphoproliferative disorders [29] (pathognomonic for IgG4-related orbital disease), to depict tumors extended to sinonasal cavity from the intracranium, to show intracranial and orbital complications from sinusitis, inflammatory polyps, oedema [28], and trauma [30].

The infraorbital canal and Haller's cells were evaluated together in edentulous patients. Therefore mean age of the study group was higher (63.63±10.113). This age range was selected to distinguish anatomic variations and undefined pain for the clinical evaluation of edentulous patients. Edentulous patients demand comfort for chewing and speaking. Furthermore, undefined pain felt in maxilla and mandible of edentulous patients may be diagnostically challenging for practitioners [20]. Longstanding edentulous alveolar ridges can atrophy and need surgical treatment for stabilization of prosthetic restoration. The recognition of radiographic imaging patterns of anatomical details is valuable for surgical planning [31].

## 5. Conclusion

To our knowledge, there has been no study in the literature assessing for both infraorbital ethmoid cells and the infraorbital canal on the edentulous patients' panoramic radiographs.

The data obtained from our study encouraged us to think that

1. future studies may be focused on Haller's cells prevalence of patients who have unexplained pain;
2. the course of infraorbital canal in the maxillary sinus and its relationship with sinus septae and Haller's cells must be studied in depth with CBCT, particularly by oral and maxillofacial radiologists and surgeons in dental practice.

## Figures and Tables

**Table 1:** Infraorbital canal (IOC) visibility sides and types.

**IOC**	Right side	Unilateral	Type 1	6(2.1%)
			Type 2	4(1.4%)
			Type 3	18(6.2%)
		Bilateral	Type 1	79(27.1%)
			Type 2	26(8.9%)
			Type 3	98(33.7%)
	Left side	Unilateral	Type 1	2(0.7%)
			Type 2	6(2.1%)
			Type 3	7(2.4%)
		Bilateral	Type 1	79(27.1%)
			Type 2	33(11.3%)
			Type 3	87(29.9%)

**Table 2:** Infraorbital ethmoid cell (Haller's Cell) types and sides.

**Haller's Cell**	Right side	Unilateral	Unilocular	20(6.9%)
			Multilocular	3(1%)
			Unilocular with septae	2(0.7%)
		Bilateral	Unilocular	16(5.5%)
			Multilocular	1(0.3%)
			Unilocular with septae	2(0.7%)
	Left side	Unilateral	Unilocular	21(7.2%)
			Multilocular	1(0.3%)
			Unilocular with septae	3(1.0%)
		Bilateral	Unilocular	16(5.5%)
			Multilocular	0(0%)
			Unilocular with septae	3(1.0%)

## Data Availability

The data used to support the findings of this study are available from the corresponding author upon request.
